# Pathogenesis and Management of Macular Hole: Review of Current Advances

**DOI:** 10.1155/2019/3467381

**Published:** 2019-05-02

**Authors:** Guzel Bikbova, Toshiyuki Oshitari, Takayuki Baba, Shuichi Yamamoto, Keisuke Mori

**Affiliations:** ^1^Department of Ophthalmology and Visual Science, Chiba University Graduate School of Medicine, Inohana 1-8-1, Chuo-ku, Chiba 260-8670, Japan; ^2^Department of Ophthalmology, International University of Health and Welfare, 537-3, Iguchi, Nasushiobara 329-2763, Tochigi, Japan

## Abstract

Macular hole has been believed to be a disorder of vitreomacular interface, which forms as a result of abnormal vitreous traction from incomplete vitreous detachment. However, our recent studies demonstrated that dynamic forces, caused by mobile posterior cortical vitreous with fluid currents, exist already at early stages of macular hole development. Therefore, in eyes with flexible vitreous, the contributions of tractional forces due to vitreous shrinkage are unlikely. These facts indicate that in the development of idiopathic macular holes, there is a greater contribution of dynamic forces than has been previously reported. This review also evaluates the recent findings in the assessment of the idiopathic macular holes and the recent therapeutic strategies for optimal management. Inner limiting membrane is considered to improve anatomical closure rate; however, it is still questionable if peeling is necessary in holes less than 250 *µ*m. There are plenty of publications indicating that in the management of small and medium size hole (less than 400 *µ*m), use of long-lasting gas and face-down position is not always required; however, it may be necessary for the treatment of large holes. Ocriplasmin and expansile gas had been reported to be successful for management of small- and medium-sized holes and vitreomacular attachment.

## 1. Introduction

Macular hole is a retinal defect located in the centre of the fovea, causing significant vision impairment [1]. Knapp in 1869 was the first who reported a macular hole with traumatic origin [2]. The term “hole in the macula” was used by Ogilvie in 1900 [3].

There are two types of macular holes which can be observed: idiopathic macular holes (IMH) [1], which is caused by vitreous traction on the foveal centre anteroposterior and tangential directions, and traumatic macular hole (TMH) usually caused by mechanic blunt injury of the eye [4]. However, in the recent literature, the term idiopathic is not used anymore, as the vitreous traction is the known reason for MH development [5].

Development of MH with retinal detachment is a specific complication of high myopia with posterior staphyloma (although in some patients with a staphyloma retinal detachment can develop without a hole) [6].

MHs can resolve, persist stable, or progress to full-thickness macular holes. According to Gass, in case if complete posterior vitreous detachment develops, the fovea can return to normal, or if Müller cell cone is stripped from the retinal surface, a lamellar hole may develop [1].

In the general population, the prevalence of MHs was reported to be around 3.3 per 1,000 people [7]. Until 1991, MH was considered to be an untreatable condition, but for the past decade, surgical techniques for closing the hole and improving the central vision are carried out as a routine practice. Vitrectomy surgery with the use of long-acting gas and postoperative positioning face-down for 1 week was the only available treatment; however, nowadays, there are a number of options to choose from. This review discusses the recent finding on the management of IMHand classification and surgical options for optimal treatment.

## 2. Classification, Evaluation, and Pathogenesis

In the MH formation, an important role is on the vitreous traction [8]. Gass classification is based on stepwise development of macular holes according to how the vitreous exerts traction on the fovea (Table 1). In 2013, the international vitreomacular traction study (IVTS) proposed anatomic classification based on OCT findings [9], in which MHs are divided into primary or secondary by cause and also by the presence or absence of vitreous attachment. Additionally, based on the horizontally measured linear width at the narrowest point of the hole, they had been classified into small (≤250 *µ*m), medium (>250 *µ*m and ≤400 *µ*m), and large (>400 *µ*m) [9]. However, in a recent publication, according to Soon et al., there is a little difference between 350 *μ*m and 450 *μ*m MH, and in sense of planning surgery, 400 *μ*m is not very practical [10]. According to their study, 650 *μ*m is a much better marker to divide medium and large macular holes, based on their results with 90% success in standard full-thickness macular hole (FTMH) vitrectomy involving internal limiting membrane (ILM) peel and gas tamponade on medium MH between 250 and 650 *μ*m [10]. They noted in their study that standard surgery for large MH (>650 *μ*m) is less successful, and such techniques as ILM flaps and retinal expansion technique for macular hole apposition (RETMA) should be considered for this matter [10]. Also, in a study of Yu et al., they conclude that stage 3 MHs, instead of smaller diameters and shorter duration of symptoms, have similar clinical and morphological features with stage 4 MHs according to Gass' classification (1995), where MHs smaller than 400 *μ*m are excluded from stage 3 compared to 1988 classification [11].

Recent publication of results of European Eye Epidemiology (E3) consortium to standardize epidemiological studies proposed a spectral-domain optical coherence tomography- (SD-OCT-) based classification for macular diseases, where MHs are subclassified as small (<250 *μ*m), medium (>250 to ≤400 *μ*m), and large macular hole (>400 *μ*m) [13]. A detailed classification with the acronym WISPERR, which includes 6 domains, width of vitreoretinal attachment, vitreoretinal interface changes, shape, pigment epithelial changes, elevation of the lowest point of vitreous attachment, and intraretinal changes separated into inner and outer retinal changes of focal vitreomacular attachment (VMA) and traction, has been suggested [14]. Chun et al. suggested to modify classification of MH based on OCT findings [12] into 2 types of MHs based on the level of preoperative tissue defects (the differences between them depended on peculiar characteristics of Müller cells in the fovea); additionally, this classification system determines closure patterns and visual outcomes after surgery [12]. MH subdivided according to the tissue defects into A type: dehiscent type, macular holes with few outer foveal tissue defects from central dehiscence (the A type is the photoreceptor retraction-dominant, in which foveal pseudocysts and intrafoveal splitting occur) and B type: tearing type, macular holes occur from substantial outer tissue loss as a result of full-thickness tearing (tractional force affects Müller cells eccentric to the centre of the foveolar floor, when traction is large and vitreofoveal adhesion in intensive). Stage 2 MHs are subdivided into 2-A and 2-B holes, where stage 1-A holes progress to stage 2-A holes, and stage 1-B holes progress to stage 2-B holes. In both cases, the anterior traction of the incompletely detached posterior hyaloid is a major factor contributing to the progression of holes from A to B type [12]. Preoperative examination should include measurements of visual acuity, metamorphopsia record by Amsler chart, slit-lamp biomicroscopy, and OCT evaluation. OCT helps not only visualize the vitreomacular traction but also to plan the surgical manoeuvres. It is very important to pay attention on the size of the holes since the size is crucial for visual prognosis and anatomical closure [12].

Some holes, especially without vitreomacular attachment (VMA), may close spontaneously. Spontaneous resolution of such holes was reported in a range from 2.7% to 8.6% of cases [15–17]. Some patients can be observed with so-called macular microholes, holes within 50–100 *µ*m without VMA [18]. But usually holes progress and 34.4%–79% [19, 20] had progression in hole size from 2 to 6 years follow up. Takahashi et al. reported that a second full-thickness MH was developed in 5 of 16 fellow eyes (31%) with a foveolar detachment and in 5 of 9 fellow eyes with a foveolar detachment and inner foveal splits [21].

SD-OCT provides precise measurements of MH dimensions, and contemporary equipment allows to obtain images with the resolution between 10 *µ*m and 25 *µ*m [22]. OCT images are very helpful for examining relationships between the retina and vitreous as well as associated structures adjacent to and outside of the macula.

Measurements of base diameter (BD) and minimum linear diameter (MLD), hole form factor, macular hole index, and tractional hole index are described for MH evaluation [23, 24].

In 2012, Mori et al. published the results of wide-angle montaged images of SD-OCT in patients with macular hole. They described two patterns of posterior vitreous configuration, “smooth or wavy” vitreous surfaces. Posterior vitreous cortex had a smooth curvature at the onset of separation, and with progressive separation, posterior vitreous folds increased. This finding indicates redundancy progression of posterior cortical vitreous through the process of MH formation. This “wavy” interface implies vitreous mobility. The mass and movement of the vitreous represents the potential force to act on the retina. In addition, granular hyperreflection was observed in 50% to 60% of eyes with stage 1 or 2 holes in the peripheral vitreous and in 33% of eyes with stage 3 or 4 holes, and also they described areas of peripheral double-layered retinoschisis in the peripheral retina adherent to posterior vitreous cortex [25].

There has been a controversy in the origin of vitreous traction in the pathogenesis of MH formation. Guyer and Green [26] and Johnson [27] suggested that dynamic tractional forces that are generated by posterior cortical vitreous movement during the rotations of the eye may play an important role in the development of MH. Mori et al. also described the mobility of posterior cortical vitreous, using the OCT tracking system. They scanned baseline and after the vertical and horizontal saccades of the same area of the fundus using eye tracking system. The tracking system of OCT enables image registration of the same area allowing longitudinal imaging. These merged images demonstrated the posterior vitreous duplication, indicating its mobility. It was reported that the incidence of cortical vitreous duplication in eyes with idiopathic MH was 92%, increasing with progressing stage of the MH [28]. Therefore, they proposed that role of dynamic forces to the development of idiopathic MH is greater than that has been thought previously.

Additionally, need to mention, even though IVTS group provided in 2013 definitions of lamellar MH and macular pseudohole based on B-scan OCT image findings [9], recent advances in OCT allowed to include such findings as lamellar hole-associated epiretinal proliferation [29]. In recent study, Romano et al. evaluated the macular pigment optical density (MPOD) by the one-wavelength fundus reflectance method, and they found statistically significant differences in MPOD between healthy eyes and eyes with vitreoretinal interface syndromes (iERM or MH) in case of MH, and they observed the lack of macular pigment in an area corresponding to the hole surface, which occurred as a result of opening of the fovea and a centrifugal displacement of the macular pigment [30].

## 3. Treatment

The most important prediction for successful MH surgery is preoperative visual acuity (VA). The better the preoperative VA is, the higher the rates of visual gain and anatomical closure [31, 32]. Short duration of symptoms is also a crucial factor for better visual outcomes and anatomical closure of MH [33]. Stage 1 MH can be spontaneously resolved in some occasions; however, they need to be under close observation [15]. Stage 2 and higher are usually indications for surgical correction, for better surgical results (anatomical and functional) [11, 15].

### 3.1. Vitrectomy

Vitrectomy for closure of MH is reported to have high success (85%–100%) [34]. Jackson et al. reported a multicentre database study of 1,045 patients, where 48.6% achieved visual success at 12 weeks postoperatively; it was increased to 58.3% at 52 weeks [35]. Herneiss et al. reported results after 1 year following pars plana vitrectomy (PPV), where macular hole closure was achieved in 57 of 59 patients (97%), and significant improvements in general vision and quality of life were reported [36].

### 3.2. Internal Limiting Membrane (ILM) Peeling

Eckardt et al. in 1997 [37] was the first to describe ILM peeling, and it was reported to give good results and increase the rate of closure for MH. Several randomized control trials confirmed the efficacy of ILM peeling in MH surgery. According to Lois et al. [38], at 1 month postoperatively in patients undergoing ILM peeling, closure was achieved in 84% compared with 48% who did not have ILM peeling (*P* < 0.001).

Nowadays, ILM peeling becomes a routine technique for MH surgery for most surgeons. For staining the ILM, such adjuvants as indocyanine green [39], triamcinolone acetonide [40], and brilliant blue G (BBG) [41] are used. Variations of ILM peel such as inverted ILM peeling and ILM-free flap are in a use for surgeons. In large MH, Michalewska et al. reported a surgical technique called inverted ILM peeling to overcome surgical failures [42]. Kuriyama et al. reported that this technique demonstrated an outstanding result in cases of MH associated with pathologic myopia [43]. According to recent randomized control trials (RCT), inverted ILM flap technique demonstrated higher anatomical success rate with a better functional outcome; however, statistically significant difference was not achieved [44]. Soon et al. reported application of ILM peeling for the management of large MH [10], and they claim 90% success with standard MH vitrectomy involving ILM peel and gas tamponade in medium MH between 250 and 650 *μ*m. Free flap ILM is used in patients with persistent MH hole after previous surgery, where a free patch of peripheral peeled ILM is placed over or in the MH [45].

As an alternative to ILM peeling ILM, abrasion had been proposed, in order to thin the ILM and loosen its adhesion to the underlying retina while still stimulating glial cell activation [46]. However, ILM peel can have negative consequences on a function (paracentral scotomas and reduced central retinal sensitivity) and retinal structure (changes in the retinal morphology such as retinal displacement); some techniques of ILM peeling and variety of dyes used may add some other risks (such as submacular RPE atrophy of glial cells response on closure, dye toxicity, etc.) [47].

### 3.3. Gas Type and Tamponade

Fluid-air exchange with subsequent gas exchange was usually carried out, after vitrectomy and ILM peeling [48]. Gas tamponade is helping in hole closure by preventing trans-hole fluid leakage from the vitreous cavity, pumping the retinal pigment epithelium in order to remove the subretinal fluid, decreased retinal oedema by reducing transretinal uveal-scleral outflow, also generating interfacial surface tension force between the retina and the gas bubble in order to pull the hole edges [49], helping glial cell to migrate for the gap closure by creating the surface [50, 51].

Different isovolumetric gas concentrations used by surgeons and total duration of gas filling usually range from 2 to 11 weeks (2–2.5 for SF6, 4–6 for C2F6, and 8–11 for C3F8) [8].

Closure rate of 90% with SF6 and 91% with C3F8 was reported in a retrospective cohort study with face-down positioning postoperatively [52]. In a prospective randomized control trial published by Briand et al., 59 patients were randomized to either SF6 or C3F8 with differing posture regimes. In those patients, closure was achieved in 93.3% and 92.9% and best-corrected visual acuity improved by 17.7 letters and 16.9 letters, respectively [53].

### 3.4. Posturing

Maintaining the face-down positioning with gas tamponade was reported to be useful in the MH closure; however, it is not comfortable for patients and can be associated with complications, such as back pain or ulnar nerve palsies [54]. However, optimal duration of posturing is still questionable. Recent publications indicate that long-lasting posturing is not necessary for MH closure after vitrectomy with ILM peeling and a long-acting gas tamponade [55–57].

OCT in the early postoperative period is reported to be a useful tool to determine whether MH is closed even in gas-filled eyes. Swept-source optical coherence tomography (SS-OCT) is known to have longer wavelength than spectral domain OCT and has the ability of a higher penetration through the opaque media, thus resulting in high-quality, high-resolution images in gas-filled eyes [58, 59]. The SS-OCT was reported to be able to determine the MH status in gas-filled eyes as early as 2 hours after surgery [60]. Sano et al. reported that use of the SS-OCT protocol can significantly decrease the duration of the face-down positioning after MH surgery, thus preventing from unnecessary posturing in eyes with a closed MH [61]. Chow and Chaudhary published their findings of an OCT-based positioning for MH surgery with daily SD-OCT imaging in 35 eyes until the MH was confirmed to be closed when face-down positioning was stopped [62]. Using OCT control for monitoring MH closure is helpful for improvement of surgical outcome and minimizing discomfort for the patients.

### 3.5. Ocriplasmin

Ocriplasmin is a truncated form of human plasmin with proteolytic activity for vitreoretinal interface components including fibronectin and laminin; it was approved for the treatment of symptomatic VMA, including association of VMA with MH of <400 [8, 15]. A single intravitreal injection of ocriplasmin (125 *µ*g) demonstrated a better resolution of macular adhesion (26.5 vs. 10.1% in the placebo group) as well as an increased rate of nonsurgical macular hole closure (40.6 vs. 10.6% in the placebo group) in a multicentre, randomized, double-blind, phase III trials [63]. Ocriplasmin applied as intravitreal injection and by activation of endogenous matrix metalloproteinase-2 resulting in precipitation of VR separation, in case of early formation of MH, can result in hole closure [64]. Ocriplasmin was reported to be safe according to phase III trials; however, some adverse effects such as floaters, photopsia, and transiently blurred vision can be observed, and those effects occur due to vitreolytic effect [65].

### 3.6. 27-Gauge Vitrectomy

27 G surgery is a technique using instruments with a diameter of instrument ∼0.35 mm. However, visual outcome and closure results were reported to be comparable for narrow-gauge surgery vs 20 G [66]. Sakaguchi et al. reported that epiretinal membrane removal surgery without vitrectomy can be performed with the 27-gauge system [67].

## 4. Conclusion

Current treatment options for MH management discussed in this review allow to achieve high rate of macular closure and improve visual recovery. Use of repeated OCT is useful for confirmation that correct tactic had been chosen particular for expansile gas or ocriplasmin usage. For the patients with small or medium holes, significantly better results are expected by application of ocriplasmin, however, with lower closure rate that surgery. Holes without VMA vitrectomy is usually the only possible treatment with the choice to perform ILM peeling and uses a short-term or long-term gas or prone posture.

## Figures and Tables

**Table 1:** Classifications of macular holes.

Gass stages	Description	OCT	IVTS group classification	E3-SD‐OCT-based classification	Chun et al. classification [12]	
					Type A: dehiscence and centrifugal retraction	Type B: tearing or full thickness fovea
Stage 0	VMA in the fellow eye of a patient with a known/previous MH without any change in foveal architecture		VMA			

Stage 1A	Impending macular hole with outer retinal elevation from RPE at foveal centre	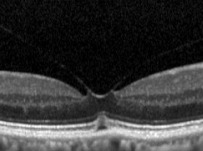	VMT without MH: can occur with outer or inner retinal changes or both		(i) Impending hole (ii) Occult hole	Occult hole

1B		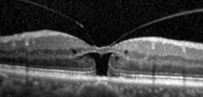				

Stage 2	≤400 *µ*m MH with VMA	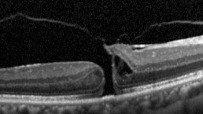	Small- or medium-sized MH with VMT	(i) Small (<250 *μ*m) (ii) Medium (>250 to ≤400 *μ*m)	Opercula that are still attached to the hole edge	Opercula that are still attached to the hole edge

Stage 3	>400 *µ*m MH without VMA	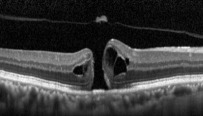	Large MH without VMT	Large macular hole (>400 *μ*m)	Small holes (i.e., <400 *μ*m) and longer lengths from the tip of the ELM to the GCL	>400 *μ*m

Stage 4	MH with complete posterior vitreous detachment	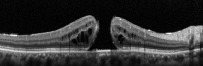	Small, medium, or large MH without VMT			

IVTS, International Vitreomacular Traction Study Group classification; E3-SD‐OCT, European Eye Epidemiology (E3) consortium spectral‐domain optical coherence tomography-based classification; VMA, vitreomacular adhesion; RPE, retinal pigment epithelium; MH, macular hole; VMT, vitreomacular traction; ELM, external limiting membrane; GCL, ganglion cell layer.

## References

[1] Gass J. D. (1988). Idiopathic senile macular hole. Its early stages and pathogenesis. *Archives of Ophthalmology*.

[2] Knapp H. (1869). About isolated ruptures of the choroid as a result of trauma to the eyeball. *Archiv fuer Augenheilkunde*.

[3] Ogilvie F. M. (1900). On one of the results of concussion injuries of the eye (“holes” at the macula). *Archive of Transactions of the American Ophthalmological Society*.

[4] Liu W., Grzybowski A. (2017). Current management of traumatic macular holes. *Journal of Ophthalmology*.

[5] Morescalchi F., Costagliola C., Gambicorti E., Duse S., Romano M. R., Semeraro F. (2017). Controversies over the role of internal limiting membrane peeling during vitrectomy in macular hole surgery. *Survey of Ophthalmology*.

[6] Ikuno Y. (2017). Overview of the complications of high myopia. *Retina*.

[7] Ezra E. (2001). Idiopathic full thickness macular hole: natural history and pathogenesis. *British Journal of Ophthalmology*.

[8] Madi H. A., Masri I., Steel D. H. (2016). Optimal management of idiopathic macular holes. *Clinical Ophthalmology*.

[9] Duker J. S., Kaiser P. K., Binder S. (2013). The international vitreomacular traction study group classification of vitreomacular adhesion, traction, and macular hole. *Ophthalmology*.

[10] Soon W. C., Patton N., Ahmed M. (2018). The manchester large macular hole study: is it time to reclassify large macular holes?. *American Journal of Ophthalmology*.

[11] Yu Y., Liang X., Wang Z., Wang J., Liu W. (2018). Clinical and morphological comparisons of idiopathic macular holes between stage 3 and stage 4. *Graefe’s Archive for Clinical and Experimental Ophthalmology*.

[12] Chung H., Byeon S. H. (2017). New insights into the pathoanatomy of macular holes based on features of optical coherence tomography. *Survey of Ophthalmology*.

[13] Gattoussi S., Buitendijk G. H., Peto T. (2018). The european eye epidemiology spectral‐domain optical coherence tomography classification of macular diseases for epidemiological studies. *Acta Ophthalmologica*.

[14] Steel D. H. W., Downey L., Greiner K. (2016). The design and validation of an optical coherence tomography-based classification system for focal vitreomacular traction. *Eye*.

[15] Oh H., Oshima Y. (2014). Microincision vitrectomy surgery. Emerging techniques and technology. *Developments in Ophthalmology*.

[16] Sugiyama A., Imasawa M., Chiba T., Iijima H. (2012). Reappraisal of spontaneous closure rate of idiopathic full-thickness macular holes. *Open Ophthalmology Journal*.

[17] Tadayoni R., Massin P., Haouchine B., Cohen D., Erginay A., Gaudric A. (2001). Spontaneous resolution of small stage 3 and 4 full-thickness macular holes viewed by optical coherence tomography. *Retina*.

[18] Reddy C. V., Folk J. C., Feist R. M. (1996). Microholes of the macula. *Archives of Ophthalmology*.

[19] Chew E. Y., Sperduto R. D., Hiller R. (1999). Clinical course of macular holes. *Archives of Ophthalmology*.

[20] Casuso L. A., Scott I. U., Flynn H. W. (2001). Long-term follow-up of unoperated macular holes. *Ophthalmology*.

[21] Takahashi A., Yoshida A., Nagaoka T. (2011). Macular hole formation in fellow eyes with a perifoveal posterior vitreous detachment of patients with a unilateral macular hole. *American Journal of Ophthalmology*.

[22] Barak Y., Ihnen M. A., Schaal S. (2012). Spectral domain optical coherence tomography in the diagnosis and management of vitreoretinal interface pathologies. *Journal of Ophthalmology*.

[23] Ip M. S., Baker B. J., Duker J. S., Reichel E., Baumal C. R. (2002). Anatomical outcomes of surgery for idiopathic macular hole as determined by optical coherence tomography. *Archives of Ophthalmology*.

[24] Ruiz-Moreno J. M., Staicu C., Piñero D. P., Montero J., Lugo F., Amat P. (2008). Optical coherence tomography predictive factors for macular hole surgery outcome. *British Journal of Ophthalmology*.

[25] Mori K., Kanno J., Gehlbach P. L., Yoneya S. (2012). Montage images of spectral-domain optical coherence tomography in eyes with idiopathic macular holes. *Ophthalmology*.

[26] Guyer D. R., Green W. R., Franklin R. M. (1993). Idiopathic macular holes and precursor lesions. *Retina and Vitreous*.

[27] Johnson M. W. (2010). Posterior vitreous detachment: evolution and complications of its early stages. *American Journal of Ophthalmology*.

[28] Mori K., Gehlbach P. L., Kishi S. (2015). Posterior vitreous mobility delineated by tracking of optical coherence tomography images in eyes with idiopathic macular holes. *American Journal of Ophthalmology*.

[29] dell’Omo R., Virgili G., Rizzo S. (2017). Role of lamellar hole-associated epiretinal proliferation in lamellar macular holes. *American Journal of Ophthalmology*.

[30] Romano M. R., Cennamo G., Grassi P., Sparnelli F., Allegrini D., Cennamo G. (2018). Changes in macular pigment optical density after membrane peeling. *PLoS One*.

[31] Jaycock P. D., Bunce C., Xing W. (2005). Outcomes of macular hole surgery: implications for surgical management and clinical governance. *Eye*.

[32] Gupta B., Laidlaw D. A., Williamson T. H., Shah S. P., Wong R., Wren S. (2011). Predicting visual success in macular hole surgery. *British Journal of Ophthalmology*.

[33] Stec L. A., Ross R. D., Williams G. A., Trese M. T., Margherio R. R., Cox M. S. (2004). Vitrectomy for chronic macular holes. *Retina*.

[34] Wakely L., Rahman R., Stephenson J. (2012). A comparison of several methods of macular hole measurement using optical coherence tomography, and their value in predicting anatomical and visual outcomes. *British Journal of Ophthalmology*.

[35] Jackson T. L., Donachie P. H. J., Sparrow J. M., Johnston R. L. (2013). United Kingdom national ophthalmology database study of vitreoretinal surgery: report 2, macular hole. *Ophthalmology*.

[36] Hirneiss C., Neubauer A. S., Gass C. A. (2007). Visual quality of life after macular hole surgery: outcome and predictive factors. *British Journal of Ophthalmology*.

[37] Eckardt C., Eckardt U., Groos S., Luciano L., Reale E. (2007). Removal of the internal limiting membrane in macular holes. Clinical and morphological findings. *Ophthalmologe*.

[38] Lois N., Burr J., Norrie J. (2011). Internal limiting membrane peeling versus no peeling for idiopathic full-thickness macular hole: a pragmatic randomized controlled trial. *Investigative Opthalmology & Visual Science*.

[39] Kadonosono K., Itoh N., Uchio E., Nakamura S., Ohno S. (2000). Staining of internal limiting membrane in macular hole surgery. *Archives of Ophthalmology*.

[40] Kimura H., Kuroda S., Nagata M. (2004). Triamcinolone acetonide-assisted peeling of the internal limiting membrane. *American Journal of Ophthalmology*.

[41] Enaida H., Hisatomi T., Hata Y. (2006). Brilliant blue G selectively stains the internal limiting membrane/brilliant blue G??? Assisted membrane peeling. *Retina*.

[42] Michalewska Z., Michalewski J., Adelman R. A., Nawrocki J. (2010). Inverted internal limiting membrane flap technique for large macular holes. *Ophthalmology*.

[43] Kuriyama S., Hayashi H., Jingami Y., Kuramoto N., Akita J., Matsumoto M. (2013). Efficacy of inverted internal limiting membrane flap technique for the treatment of macular hole in high myopia. *American Journal of Ophthalmology*.

[44] Kannan N. B., Kohli P., Parida H., Adenuga O. O., Ramasamy K. (2018). Comparative study of inverted internal limiting membrane (ILM) flap and ILM peeling technique in large macular holes: a randomized-control trial. *BMC Ophthalmology*.

[45] Wong D., Steel D. H. W. (2016). Free ILM patch transplantation for recalcitrant macular holes; should we save some internal limiting membrane for later?. *Graefe’s Archive for Clinical and Experimental Ophthalmology*.

[46] Mahajan V. B., Chin E. K., Tarantola R. M. (2015). Macular hole closure with internal limiting membrane abrasion technique. *JAMA Ophthalmology*.

[47] Chatziralli I. P., Theodossiadis P. G., Steel D. H. W. (2018). Internal limiting membrane peeling in macular hole surgery; why, when, and how?. *Retina*.

[48] Kelly N. E., Wendel R. T. (1991). Vitreous surgery for idiopathic macular holes. *Retina*.

[49] Chang S., Reppucci V., Zimmerman N. J., Heinemann M.-H., Coleman D. J. (1989). Perfluorocarbon liquids in the management of traumatic retinal detachments. *Ophthalmology*.

[50] Smiddy W. E., Flynn H. W. (2004). Pathogenesis of macular holes and therapeutic implications. *American Journal of Ophthalmology*.

[51] Thompson J. T., Smiddy W. E., Glaser B. M., Sjaarda R. N., Flynn H. W. (1996). Intraocular tamponade duration and success of macular hole surgery. *Retina*.

[52] Kim S. S., Smiddy W. E., Feuer W. J., Shi W. (2008). Outcomes of sulfur hexafluoride (SF6) versus perfluoropropane (C3F8) gas tamponade for macular hole surgery. *Retina*.

[53] Briand S., Chalifoux E., Tourville E. (2015). Prospective randomized trial: outcomes of SF6 versus C3F8 in macular hole surgery. *Canadian Journal of Ophthalmology*.

[54] Yamashita T., Sakamoto T., Yamashita T. (2014). Individualized, spectral domain-optical coherence tomography-guided facedown posturing after macular hole surgery. *Retina*.

[55] Heath G., Rahman R. (2010). Combined 23-gauge, sutureless transconjunctival vitrectomy with phacoemulsification without face down posturing for the repair of idiopathic macular holes. *Eye*.

[56] Nadal J., Delas B., Piñero A. (2012). Vitrectomy without face-down posturing for idiopathic macular holes. *Retina*.

[57] Iezzi R., Kapoor K. G. (2013). No face-down positioning and broad internal limiting membrane peeling in the surgical repair of idiopathic macular holes. *Ophthalmology*.

[58] Choma M. A., Hsu K., Izatt J. A. (2005). Swept source optical coherence tomography using an all-fiber 1300-nm ring laser source. *Journal of Biomedical Optics*.

[59] Ohno-Matsui K., Akiba M., Moriyama M., Ishibashi T., Tokoro T., Spaide R. F. (2011). Imaging retrobulbar subarachnoid space around optic nerve by swept-source optical coherence tomography in eyes with pathologic myopia. *Investigative Opthalmology & Visual Science*.

[60] Kikushima W., Imai A., Toriyama Y., Hirano T., Murata T., Ishibashi T. (2015). Dynamics of macular hole closure in gas- filled eyes within 24 h of surgery observed with swept source optical coherence tomography. *Ophthalmic Research*.

[61] Sano M., Inoue M., Itoh Y. (2017). Duration of prone positioning after macular hole surgery determined by swept-source optical coherence tomography. *Retina*.

[62] Chow D. R., Chaudhary K. M. (2015). Optical coherence tomography-based positioning regimen for macular hole surgery. *Retina*.

[63] Singh R. P., Li A., Bedi R. (2014). Anatomical and visual outcomes following ocriplasmin treatment for symptomatic vitreomacular traction syndrome. *British Journal of Ophthalmology*.

[64] Takano A., Hirata A., Inomata Y. (2005). Intravitreal plasmin injection activates endogenous matrix metalloproteinase-2 in rabbit and human vitreous. *American Journal of Ophthalmology*.

[65] Kaiser P. K., Kampik A., Kuppermann B. D., Girach A., Rizzo S., Sergott R. C. (2015). Safety profile of ocriplasmin for the pharmacologic treatment of symptomatic vitreomacular adhesion/traction. *Retina*.

[66] Krishnan R., Tossounis C., Fung Yang Y. (2013). 20-gauge and 23-gauge phacovitrectomy for idiopathic macular holes: comparison of complications and long-term outcomes. *Eye*.

[67] Sakaguchi H., Oshima Y., Tano Y. (2007). 27-gauge transconjunctival nonvitrectomizing vitreous surgery for epiretinal membrane removal. *Retina*.

